# Draft Genome Sequences of Five *Enterococcus* Species Isolated from the Gut of Patients with Suspected *Clostridium difficile* Infection

**DOI:** 10.1128/genomeA.00379-17

**Published:** 2017-05-18

**Authors:** Eduardo Castro-Nallar, Sandro L. Valenzuela, Sebastián Baquedano, Carolina Sánchez, Fabiola Fernández, Annette N. Trombert

**Affiliations:** a Center for Bioinformatics and Integrative Biology, Facultad de Ciencias Biológicas, Universidad Andrés Bello, Santiago, Chile; b Escuela de Tecnología Médica, Facultad de Ciencias, Universidad Mayor, Huechuraba, Santiago, Chile; c Centro de Genómica y Bioinformática, Facultad de Ciencias, Universidad Mayor, Santiago, Chile

## Abstract

We present draft genome sequences of five *Enterococcus* species from patients suspected of *Clostridium difficile* infection. Genome completeness was confirmed by presence of bacterial orthologs (97%). Gene searches using Hidden-Markov models revealed that the isolates harbor between seven and 11 genes involved in antibiotic resistance to tetracyclines, beta-lactams, and vancomycin.

## GENOME ANNOUNCEMENT

Numerous reports link microbial compositional changes (dysbiosis) in the human gut microbiota to diverse disease states ranging from inflammatory illness to psychiatric conditions (1–4). In particular, studies in human and animal models have shown the importance of the gut microbiota’s capability of providing colonization resistance against *C. difficile* (5, 6). Consequently, this announcement is part of a larger project aimed at characterizing the microbiota of individuals infected by *C. difficile* in Chile, both in terms of individual isolates and microbiota compositions.

We collected fecal samples from patients suspected of being infected by *C. difficile* that presented aqueous diarrhea associated with antimicrobial drug intake. We plated samples on blood agar medium (Merck; anaerobic conditions) and grew colonies in Brucella broth at 37°C without agitation (7). For DNA extraction, we used the Wizard genomic DNA purification kit (Promega) following the manufacturer’s instructions. DNA was quantified in a fluorimeter (Qubit; Invitrogen) and its integrity was checked by agarose gel electrophoresis. We prepared sequencing libraries as in the TruSeq nano DNA LT kit (Illumina) using an average insert size of 450 bp.

We obtained between 1.7 and 2.3 million paired-end reads that we subsequently filtered to allow no undetermined bases and an average quality score per read of > Q20. We also trimmed the 5′ and 3′ ends to remove bases with quality scores of < Q20. The resulting reads were *de novo* assembled using a De Bruijn graph strategy as implemented in SPAdes 3.8 (8). Genome coverage was 48 to 173× (median = 109×). We interrogated the resulting contigs for evidence of contamination using the GenomePeek web server and found no evidence of contaminating DNA (9). We annotated the assembled genome sequences using the NCBI Prokaryotic Genome Annotation Pipeline (released 2013) (10). Information about the Pipeline can be found here: https://www.ncbi.nlm.nih.gov/genome/annotation_prok/. Additionally, genome completeness was confirmed by BUSCO analysis of prokaryotic orthologs, where we found 97% of bacteria-wide orthologs present (11). Current standards suggest that genomes with > 85% orthologs present are considered high-quality genomes (reference *E. faecium* was 93% complete; accession no. NC_017960.1) (12).

Of the five genome sequences presented here, only two were classified as known multilocus sequence types (97-19_S17 and 97-7_S6 as ST262 and ST822, respectively) (13). However, all strains were found to carry antibiotic resistance genes including *vanR* and *vanS* genes (ARO:3000574; ARO:3000071), *vanX*, and *vanY* genes (ARO:3000011; ARO:3000077), class B beta-lactamase (ARO:3000004), antibiotic efflux pumps (ARO:0010001), and tetracycline resistance genes (ARO:3000186; ARO:3000194; ARO:3000190; ARO:3000192; ARO:3000239; ARO:0000002), among others (14). This report highlights the need for comprehensive open genomic reference databases of human gut members to better address scientific questions regarding epidemiology, virulence and pathogenicity, and drug resistance. All five genomic sequences are compliant with the MIGS package “cultured bacteria/archaea, human-associated; version 4.0” (15).

### Accession number(s).

The whole-genome shotgun projects have been deposited in GenBank under the accession numbers provided in Table 1. The versions described in this paper are the first versions.

**Table 1:** Whole-genome shotgun projects accession numbers

Species	Strain	Source	Genome size (Mb)	No. of coding sequences	Accession no.	Assembly
*Enterococcus faecium*	97-19_S17	Human feces	2.7	2,915	MRYE00000000	GCA_001990575.1
*Enterococcus faecium*	97-3_S3	Human feces	2.9	3,165	MRYF00000000	GCA_001990605.1
*Enterococcus faecium*	97-6_S5	Human feces	2.5	2,698	MRYG00000000	GCA_001990565.1
*Enterococcus faecium*	97-7_S6	Human feces	3.1	3,390	MRYH00000000	GCA_001990615.1
*Enterococcus mundtii*	CGB1038-1_S1	Human feces	3.3	3,150	MSTR00000000	GCA_001990645.1

## References

[1] Knoll RL, Forslund K, Kultima JR, Meyer CU, Kullmer U, Sunagawa S, Bork P, Gehring S 30 12 2016 Gut microbiota differs between children with inflammatory bowel disease and healthy siblings in taxonomic and functional composition—a metagenomic analysis. Am J Physiol Gastrointest Liver Physiol. doi:10.1152/ajpgi.00293.2016. 28039159

[2] Castro-Nallar E, Bendall ML, Pérez-Losada M, Sabuncyan S, Severance EG, Dickerson FB, Schroeder JR, Yolken RH, Crandall KA 2015 Composition, taxonomy and functional diversity of the oropharynx microbiome in individuals with schizophrenia and controls. PeerJ 3:e1140. doi:10.7717/peerj.1140. 26336637 PMC4556144

[3] Cani PD, Bibiloni R, Knauf C, Waget A, Neyrinck AM, Delzenne NM, Burcelin R 2008 Changes in gut microbiota control metabolic endotoxemia-induced inflammation in high-fat diet-induced obesity and diabetes in mice. Diabetes 57:1470–1481. 18305141 10.2337/db07-1403

[4] Severance EG, Yolken RH, Eaton WW 2014 Autoimmune diseases, gastrointestinal disorders and the microbiome in schizophrenia: more than a gut feeling. Schizophr Res 159:14–19. doi:10.1016/j.schres.2014.07.053. 25034760 PMC4294997

[5] Britton RA, Young VB 2014 Role of the intestinal microbiota in resistance to colonization by *Clostridium difficile*. Gastroenterology 146:1547–1553. doi:10.1053/j.gastro.2014.01.059. 24503131 PMC3995857

[6] Buffie CG, Pamer EG 2013 Microbiota-mediated colonization resistance against intestinal pathogens. Nat Rev Immunol 13:790–801. doi:10.1038/nri3535. 24096337 PMC4194195

[7] Bhardwaj S, Dhawale KBJ, Patil M, Divase S 2013 *Enterococcus faecium* and *Enterococcus faecalis*, the nosocomial pathogens with special reference to multi-drug resistance and phenotypic characterization. Int J Pharm Sci Pract 2:1–10.

[8] Bankevich A, Nurk S, Antipov D, Gurevich AA, Dvorkin M, Kulikov AS, Lesin VM, Nikolenko SI, Pham S, Prjibelski AD, Pyshkin AV, Sirotkin AV, Vyahhi N, Tesler G, Alekseyev MA, Pevzner PA 2012 SPAdes: a new genome assembly algorithm and its applications to single-cell sequencing. J Comput Biol 19:455–477. doi:10.1089/cmb.2012.0021. 22506599 PMC3342519

[9] McNair K, Edwards RA 2015 GenomePeek—an online tool for prokaryotic genome and metagenome analysis. PeerJ 3:e1025. doi:10.7717/peerj.1025. 26157610 PMC4476108

[10] Tatusova T, DiCuccio M, Badretdin A, Chetvernin V, Nawrocki EP, Zaslavsky L, Lomsadze A, Pruitt KD, Borodovsky M, Ostell J 2016 NCBI prokaryotic genome annotation pipeline. Nucleic Acids Res 44:6614–6624. 27342282 10.1093/nar/gkw569 PMC5001611

[11] Simão FA, Waterhouse RM, Ioannidis P, Kriventseva EV, Zdobnov EM 2015 BUSCO: assessing genome assembly and annotation completeness with single-copy orthologs. Bioinformatics 31:3210–3212. doi:10.1093/bioinformatics/btv351. 26059717

[12] Bradnam KR, Fass JN, Alexandrov A, Baranay P, Bechner M, Birol I, Boisvert S, Chapman JA, Chapuis G, Chikhi R, Chitsaz H, Chou WC, Corbeil J, Del Fabbro C, Docking TR, Durbin R, Earl D, Emrich S, Fedotov P, Fonseca NA, Ganapathy G, Gibbs RA, Gnerre S, Godzaridis E, Goldstein S, Haimel M, Hall G, Haussler D, Hiatt JB, Ho IY, Howard J, Hunt M, Jackman SD, Jaffe DB, Jarvis ED, Jiang H, Kazakov S, Kersey PJ, Kitzman JO, Knight JR 2013 Assemblathon 2: Evaluating de novo methods of genome assembly in three vertebrate species. GigaScience 2:10. doi:10.1186/2047-217X-2-10. 23870653 PMC3844414

[13] Buultjens AH, Lam MM, Ballard S, Monk IR, Mahony AA, Grabsch EA, Grayson ML, Pang S, Coombs GW, Robinson JO 2016 Evolutionary origins of the emergent ST796 clone of vancomycin resistant *Enterococcus faecium*. PeerJ 5:e2916. doi:10.7717/peerj.2916. PMC5267571 28149688

[14] Gibson MK, Forsberg KJ, Dantas G 2015 Improved annotation of antibiotic resistance determinants reveals microbial resistomes cluster by ecology. ISME J 9:207–216. doi:10.1038/ismej.2014.106. 25003965 PMC4274418

[15] Field D, Garrity G, Gray T, Morrison N, Selengut J, Sterk P, Tatusova T, Thomson N, Allen MJ, Angiuoli SV, Ashburner M, Axelrod N, Baldauf S, Ballard S, Boore J, Cochrane G, Cole J, Dawyndt P, De Vos P, DePamphilis C, Edwards R, Faruque N, Feldman R, Gilbert J, Gilna P, Glöckner FO, Goldstein P, Guralnick R, Haft D, Hancock D, Hermjakob H, Hertz-Fowler C, Hugenholtz P, Joint I, Kagan L, Kane M, Kennedy J, Kowalchuk G, Kottmann R, Kolker E 2008 The minimum information about a genome sequence (MIGS) specification. Nat Biotechnol 26:541–547. doi:10.1038/nbt1360. 18464787 PMC2409278

